# Outcomes of diagnostic and therapeutic EUS procedures in live course *versus* routine service between 2015 and 2021: A propensity score analysis

**DOI:** 10.1097/eus.0000000000000172

**Published:** 2026-03-19

**Authors:** Tiffany Y. Chua, Anthony Y. B. Teoh, Shannon M. Chan, Terry C. F. Yip, Ting Ting Chan, James Y. W. Lau, Philip W. Y. Chiu, Raymond S. Y. Tang

**Affiliations:** 1 Institute of Digestive Disease, The Chinese University of Hong Kong, Hong Kong, China; 2 Division of Gastroenterology, Hepatology, and Nutrition, University of Florida, Gainesville, FL, USA; 3 Department of Surgery, The Chinese University of Hong Kong, Hong Kong, China; 4 Department of General Surgery, Hong Kong Sanatorium Hospital, Hong Kong, China; 5 Medical Data Analytics Centre, The Chinese University of Hong Kong, Hong Kong, China; 6 Li Ka Shing Institute of Health Sciences, The Chinese University of Hong Kong, Hong Kong, China; 7 Department of Medicine and Therapeutics, The Chinese University of Hong Kong, Hong Kong, China.

**Keywords:** live endoscopy, live courses and workshops, EUS, interventional EUS

## Abstract

**Background and Objectives:**

Live courses are important sources of endoscopy education. While diagnostic and therapeutic EUS procedures have been increasingly performed in live courses recently, data on outcomes of EUS in live courses remain scarce. This study aims to compare outcomes of EUS procedures in live courses *versus* routine clinical service.

**Methods:**

All diagnostic and therapeutic EUS cases performed in workshops at Prince of Wales Hospital at The Chinese University of Hong Kong from 2015 to 2021 were considered for inclusion. Therapeutic EUS cases were divided into 5 subtypes: rendezvous, access (biliary, pancreatic, and enteric), endohepatology, lesion ablation or injection, and drainage (cystogastrostomy and gallbladder drainage). Propensity score matching of procedure subtype, procedure date, and age-adjusted Charlson Comorbidity Index was performed to match live course and routine service cases on a 1:1 basis for comparison.

**Results:**

After propensity score matching, 108 live courses and 108 routine service cases were included in the analysis. Access procedures were the most common therapeutic EUS subtype performed, followed by lesion injection/ablation and drainage procedures. Rates of benign and malignant indications for procedure were comparable in both cohorts. Technical success was 100% in diagnostic EUS in both groups. There was no significant difference in technical success in therapeutic EUS (96.7% *vs.* 100%, *P* = 0.496). Intraprocedure and postprocedure adverse events were also comparable in both groups. There were no 30-day procedure-related mortalities.

**Conclusion:**

In this study, diagnostic and therapeutic EUS performed during live courses yield similar technical success and safety outcomes compared with cases performed routinely.

## INTRODUCTION

Live endoscopy courses or workshops that broadcast procedures while they are performed play an invaluable role in endoscopic education. Live endoscopy also serves to connect experts to trainees and endoscopic communities around the globe. While alternatives such as simulators have been demonstrated to be useful in endoscopic education, they cannot replace the opportunity to observe complex clinical decision-making, response to patient factors, and unexpected events in real time. Recordings of procedures are often edited and may not adequately convey the unpredictability inherent to clinical practice.

Endoscopic procedures featured in live courses frequently include interventional endoscopy; these cases are often more complex and confer both higher risk and benefit to the patient. Prior studies comparing outcomes in endoscopic retrograde cholangiopancreatography (ERCP) have yielded variable results, though there is no definitive evidence showing higher complication rates in live endoscopy.^[1–3]^ In the past few years, indications for EUS have rapidly expanded, transitioning from a primarily diagnostic modality to a procedure with multiple therapeutic applications ranging from drainage to endohepatology. This rapid growth has led to a corresponding increase in EUS procedures performed in therapeutic endoscopy workshops. As data on outcomes of diagnostic and therapeutic EUS performed in live courses remain scarce, we aim to compare outcomes of EUS procedures in live endoscopy workshops *versus* routine clinical service.

## METHODS

### Study design and study population

This was a retrospective comparative study using propensity score analysis. The study protocol was approved by the Joint Chinese University of Hong Kong–New Territories East Cluster Clinical Research Ethics Committee. All diagnostic and therapeutic EUS cases performed in live endoscopy workshops and in routine service at Prince of Wales Hospital at The Chinese University of Hong Kong from January 2015 to December 2021 were considered eligible for inclusion. Diagnostic cases included EUS with or without fine needle tissue acquisition and contrast-enhanced EUS. Therapeutic EUS cases were divided into 5 subtypes based on the 2021 American Gastroenterological Association white paper and similarities in endoscopic technique: rendezvous, access (transmural biliary/pancreatic duct drainage, gastroenterostomy), endohepatology (portal pressure gradient measurement), lesion ablation or injection (celiac plexus neurolysis, alcohol or radiofrequency ablation of tumors, variceal injection therapy, and fiducial marker placement), and drainage (pancreatic fluid collection drainage and gallbladder drainage).^[4]^ Ampullectomy and sessions during which ERCP was also performed outside the context of a rendezvous procedure were excluded.

### Study outcomes

Outcomes of interest included technical success, intraprocedure adverse events, postprocedure adverse events, need for reinterventions, unplanned admissions, and 30-day mortality rates.

### Statistical analysis

Due to the multiple subtypes of EUS procedures and variability in patient comorbidities, propensity score matching (PSM) was used to improve comparability of the live course and routine groups. PSM was performed for the following covariates: procedure subtype, procedure date (within 12 months), and age-adjusted Charlson Comorbidity Index within 2 points. Patients were individually matched on a 1:1 basis using nearest neighbor matching and a caliper width equal to 0.2 of the standard deviation of the logit of the propensity score. A subgroup analysis was performed to compare overseas *versus* local endoscopists in the live course group. Statistical analyses were performed using SPSS software (version 28.0, IBM, Armonk, NY, USA) and R statistical software (version 4.2.0, R Core Team 2022, Vienna, Austria). Continuous variables were reported as mean with standard deviation and compared by *t* test. Differences in categorical variables were analyzed using chi-square tests or Fisher’s exact tests. *P* values <0.05 were considered statistically significant.

## RESULTS

### Patient and procedure characteristics

Between 2015 and 2021, 125 live course and 4929 routine service EUS procedures were performed. After 1:1 PSM, a total of 216 EUS procedures were included for outcome analysis (108 live course and 108 routine). Baseline characteristics of patients and EUS procedures performed in live courses and as routine cases before and after PSM are listed in Table 1. There was no significant difference in patient demographics, Charlson Comorbidity Index, EUS procedure types, and indications after PSM. Rates of benign and malignant indications for procedures were comparable in both cohorts (*P* = 0.785). Access procedures were the most common therapeutic EUS subtype performed, followed by lesion injection/ablation and drainage procedures. There was an almost even split of local *versus* overseas invited faculty in the workshop group (49.1% *vs.* 50.9%).

**Table 1:** Baseline characteristics of patients and EUS procedures performed in the live course and the control group before and after propensity score matching for procedure type, procedure date (within 12 months), and age-adjusted Charlson Comorbidity Index.

	Before propensity score matching			After propensity score matching		
	Live course group *n* = 125	Control group *n* = 4929	*P* value	Live course group *n* = 108	Control group *n* = 108	*P* value
Age (yr), mean ± SD	67.6 ± 11.5	66.3 ± 13.1	0.213	68.1 ± 11.2	65.4 ± 13.5	0.103
Sex, male, *n* (%)	82 (65.6)	3104 (63.0)		70 (48.6)	74 (51.4)	0.564
Age-adjusted Charlson Comorbidity Index, mean ± SD	5.1 ± 2.5	5.6 ± 2.8	0.151	5.1 ± 2.5	5.1 ± 2.6	1.00
Diagnostic EUS, *n* (%)*	59 (47.2)	4576 (92.8)	<0.001	47 (43.5)	47 (43.5)	1.00
Therapeutic EUS, *n* (%)	66 (52.8)	353 (7.2)		61 (56.5)	61 (56.5)	
Rendezvous	5 (7.6)	31 (8.8)	<0.001	5 (4.6)	5 (4.6)	1.00
Access†	26 (39.4)	111 (31.4)		24 (22.2)	24 (22.2)	
Endohepatology‡	1 (1.5)	10 (2.8)		1 (0.9)	1 (0.9)	
Lesion ablation/injection§	17 (25.8)	126 (35.7)		16 (14.8)	16 (14.8)	
Drainage¶	17 (25.8)	75 (21.2)		15 (13.9)	15 (13.9)	
Indication of EUS, *n* (%)						
Benign	69 (55.2)	3617 (73.4)	<0.001	56 (51.9)	54 (50.0)	0.785
Malignancy-related	56 (44.8)	1312 (26.6)		52 (49.1)	54 (50.0)	
Endoscopist type, *n* (%)						
Local	61 (48.8)	4929 (100)	-	53 (49.1)	108 (100)	-
Overseas	64 (51.2)	N/A	-	55 (50.9)	N/A	-
Elective *vs.* urgent admission, *n* (%)						
Outpatient/elective admission	94 (75.2)	4305 (87.3)	<0.001	81 (75.0)	69 (63.9)	0.076
Urgent admission from emergency room	31 (24.8)	624 (12.7)		27 (25.0)	39 (36.1)	

* Contrast-enhanced EUS, fine needle aspiration, fine needle biopsy.

† Transmural biliary/pancreatic duct drainage, gastroenterostomy.

‡ Portal pressure measurement.

§ Celiac plexus neurolysis, alcohol or radiofrequency ablation of tumors, variceal injection therapy, fiducial marker placement.

¶ Cyst-gastrostomy, pancreatic fluid collection drainage, gallbladder drainage.

N/A, not applicable; SD, standard deviation.

### Outcome measures

#### Live course *versus* routine procedures

In this study, 43.5% of the EUS procedures were diagnostic whereas 56.5% of the EUS procedures were therapeutic in both groups. Technical success was 100% in diagnostic EUS in both workshop and routine groups. There was no significant difference in technical success when adjusted for therapeutic EUS procedures (96.7% *vs.* 100%, *P* = 0.496). Intraprocedure and postprocedure adverse events were also comparable in both groups (7.4% *vs.* 9.3%, *P* = 0.806, and 7.4% *vs.* 5.6%, *P* = 0.783, respectively) [Table 2]. Four patients in each group (3.7%) required follow-up endoscopy for an adverse event. Seven of the 8 patients underwent therapeutic EUS procedures. The remaining patient underwent a diagnostic EUS for a malignancy-related indication. No patient required interventional radiology or surgical intervention for management. There was no 30-day procedure-related mortality and no difference in 30-day all-cause mortality (54.6% *vs.* 48.1%, *P* = 0.414).

**Table 2:** Clinical outcomes of the live course and the control groups after matching for procedure type, procedure date (within 12 months), and age-adjusted Charlson Comorbidity Index.

	Live course group *n* = 108	Control group *n* = 108	*P* value
Overall technical success, *n* (%)	106 (98.1)	108 (100)	0.498
Diagnostic EUS	47/47 (100)	47/47 (100)	1.000
Therapeutic EUS	59/61 (96.7)	61/61 (100)	0.496
Overall intraprocedure adverse events, *n* (%)	8 (7.4)	10 (9.3)	0.806
Stent misdeployment	1 (0.9)	6 (5.6)	0.119
Wire shear/loss	3 (2.8)	2 (1.9)	1.000
Repeat puncture	1 (0.9)	5 (4.6)	0.212
Bleeding	2 (1.9)	2 (1.9)	1.000
Perforation	1 (0.9)	0 (0)	1.000
Overall postprocedure adverse events, *n* (%)	8 (7.4)	6 (5.6)	0.783
Perforation	1 (0.9)	0 (0)	1.000
Bleeding	3 (2.8)	1 (0.9)	0.621
Sepsis	4 (3.7)	4 (3.7)	1.000
Acute pancreatitis	0 (0)	1 (0.9)	1.000
Stent migration	1 (0.9)	2 (1.9)	0.829
Overall adverse events requiring interventions, *n* (%)	4 (3.7)	4 (3.7)	1.000
Endoscopy	4 (3.7)	4 (3.7)	1.000
Interventional radiology	0 (0)	0 (0)	-
Surgery	0 (0)	0 (0)	-
Overall 30-d procedure-related unplanned admission, *n* (%)	2 (1.9)	2 (1.9)	1.000
Overall 30-d procedure-related mortality, *n* (%)	0 (0)	0 (0)	-
Overall 30-d all-cause mortality, *n* (%)	59 (54.6)	52 (48.1)	0.414
Malignancy	37 (34.3)	37 (34.3)	0.408
Other medical conditions	22 (20.3)	15 (13.8)	

#### Subgroup analysis of overseas *versus* local endoscopists in the live course group

In the live course group, 55 cases were performed by overseas endoscopists, whereas 53 cases were performed by local endoscopists. Further matching for procedure characteristics was not performed due to the small sample size within each endoscopist group. There were no significant differences between the overall technical success (96.4% *vs.* 100%, *P* = 0.496), diagnostic EUS technical success (100% *vs.* 100%, *P* = 1.000), and therapeutic EUS technical success (93.8% *vs.* 100%, *P* = 0.493) between the 2 endoscopist groups [Table 3]. There were slightly higher intraprocedure (10.9% *vs.* 3.8%, *P* = 0.271) and postprocedure (12.7% *vs.* 1.9%, *P* = 0.061) adverse events in the overseas endoscopist group. The rates of adverse events requiring further interventions were similar between the 2 groups (5.5% *vs.* 1.9%, *P* = 0.618). There was no 30-day procedure-related mortality in both groups.

**Table 3:** Clinical outcomes of EUS procedures between overseas endoscopists and local endoscopists in the live course group.

	Overseas endoscopist group *n* = 55	Local endoscopist group *n* = 53	*P* value
Overall technical success, *n* (%)	53/55 (96.4)	53/53 (100)	0.496
Diagnostic EUS	23/23 (100)	24/24 (100)	1.000
Therapeutic EUS	30/32 (93.8)	29/29 (100)	0.493
Overall intraprocedure adverse events, *n* (%)	6/55 (10.9)	2/53 (3.8)	0.271
Stent misdeployment	0 (0)	1/53 (1.9)	0.491
Wire shear/loss	3/55 (5.5)	0 (0)	0.243
Repeat puncture	0 (0)	1/53 (1.9)	0.491
Bleeding	2/55 (3.6)	0 (0)	0.496
Perforation	1/55 (1.8)	0/53 (0)	1.000
Overall postprocedure adverse events, *n* (%)	7/55 (12.7)	1/53 (1.9)	0.061
Perforation	1/55 (1.8)	0/53 (0)	1.000
Bleeding	2/55 (3.6)	1/53 (1.9)	1.000
Sepsis	4/55 (7.3)	0/55 (0)	0.118
Acute pancreatitis	0 (0)	0 (0)	-
Stent migration	0 (0)	0 (0)	-
Overall adverse events requiring interventions, *n* (%)	3/55 (5.5)	1/53 (1.9)	0.618
Endoscopy	3/55 (5.5)	1/53 (1.9)	0.618
Interventional radiology	0 (0)	0 (0)	-
Surgery	0 (0)	0 (0)	-
Overall 30-d procedure-related unplanned admission, *n* (%)	1/55 (1.8)	1/53 (1.9)	1.000
Overall 30-d procedure-related mortality, *n* (%)	0 (0)	0 (0)	-

## DISCUSSION

While the unique educational value offered by live endoscopy workshops is well recognized, patient safety and care remain the priority. Both the American Society for Gastrointestinal Endoscopy and European Society of Gastrointestinal Endoscopy have provided guidance on best practices during these events, including the presence of a patient advocate to prioritize patient safety and privacy.^[5,6]^ Data on live endoscopy course outcomes are limited at present: prior studies primarily focused on ERCP as the procedure of interest. A study of colon endoscopic mucosal resection of large, nonpedunculated polyps (≥20 mm) indicated that a live audience did not result in more adverse events.^[7]^ Another study reported similar procedure times and *en bloc* and curative resection rates in live demonstrations of endoscopic submucosal dissection; complication rates were not evaluated owing to the smaller sample size and relative rarity of endoscopic submucosal dissection.^[8]^

To our knowledge, this is the first study examining EUS outcomes in live endoscopy workshops. We anticipate increasing inclusion of interventional EUS, in particular, as indications for this procedure continue to grow, and our data may help provide guidance on optimizing safety. Overall, our results showed no significant difference in procedural outcomes and adverse events for both diagnostic and therapeutic EUS in live workshop settings compared with routine endoscopy. Strengths of our study include the large total number of live cases and inclusion of a great variety of cutting-edge procedures: a little over half were therapeutic EUS cases. Our tertiary care center also has a high volume of EUS cases, enabling PSM and controlling for variables that may impact procedural outcomes, such as age and comorbidities.

We opted for the inclusion of diagnostic EUS procedures in our study. Diagnostic EUS carries minimal additional risk compared with esophagogastroduodenoscopy and is considered a relatively safe and low-risk procedure in experienced hands. These cases could thus more clearly demonstrate the presence or lack of additional risk in the live setting. Furthermore, inclusion of diagnostic EUS is more consistent with daily practice: even in high-volume tertiary centers, multiple higher-risk therapeutic EUS procedures are rarely performed on a single day.

In our comparison of overseas *versus* local endoscopists in the live course setting, we did observe a slight trend toward higher adverse events in the overseas endoscopist group. However, its implications are limited by the lack of PSM in this subgroup analysis. PSM was not deemed to be meaningful in view of the relatively small sample size within each endoscopist group. Technical success and need for additional endoscopic procedures were similar. This suggests that both overseas and local endoscopists may be equally prone to “stage fright” during live courses but these risks can be safely mitigated by the measures outlined in society guidelines.

Limitations exist in our study. First, while the technical success and safety profile of EUS procedures in live courses were comparable to those of routine service EUS procedures, these were data from a single, university-affiliated center. Nevertheless, since most live endoscopy courses are organized by tertiary centers and experienced advanced endoscopists, we believe findings in our study are reasonably representative and can shed light on what to expect in live EUS workshops organized by tertiary centers. Second, while a great variety of contemporary therapeutic EUS procedure subtypes were included in the current study, there were relatively fewer case numbers in the endohepatology and rendezvous procedure subtypes. However, from a practical point of view, this variation of procedure subtypes in live endoscopy courses is not uncommon since the actual procedure subtypes being demonstrated in live workshops often depend on the availability of suitable patients with relevant pathologies and faculty expertise at the time of the course conduction. Third, since the procedure times were not routinely recorded in all service cases during the study period, comparison of procedure times between live course cases and routine service cases was not feasible in this retrospective study. While one may speculate the possibility of potentially longer procedure times in live course EUS cases, the procedure times of routine service EUS cases in many teaching hospitals may not always be shorter due to the time used in teaching advanced endoscopy trainees by the supervising attending endoscopists for both diagnostic and therapeutic EUS procedures. Further studies with prospectively recorded procedure times of both routine and live course procedures would be needed to better address this question. Fourth, while the operator’s level of EUS expertise is generally anticipated to impact procedure outcomes, incorporation of the operators’ years of EUS experience as a matching covariate between the 2 groups may not be feasible in our cohorts since almost all of the routine service EUS cases had variable degree of advanced endoscopy trainee involvement in our center, which is a university-affiliated public teaching hospital. If the advanced endoscopy trainee’s years of EUS experience is used as a matching covariate, essentially it will be nearly impossible to find a matching case in the live course cohort since the live course procedures were all performed by attending endoscopists who will always have more EUS experience than the trainees. On the other hand, if the supervising attending endoscopist’s years of EUS experience in the routine service is used as the matching covariate, it is conceivable that the operator’s years of EUS experience between the 2 cohorts would be similar, making the incorporation of this covariate in the PSM less meaningful. Nevertheless, we believe the impact of this potential confounder should be relatively small in our current study because: (1) in the routine service cases, advanced endoscopy trainees performing the procedures were always under direct close supervision by the attending endoscopists who would promptly provide back up and/or take over the procedure when necessary, and (2) similarly high overall technical success and similarly low overall intraprocedure/postprocedure adverse event rates were observed between the live course cohort and the routine service cohort in the current PSM analysis without incorporating the covariate of operator’s years of EUS experience.

## CONCLUSION

In this comparative study with propensity score analysis, our data suggest that diagnostic and therapeutic EUS performed during a live course yield similar technical success and safety outcomes compared with cases performed routinely. This reassuring data provides evidence to justify continued conduction of EUS during live courses for education.

## Acknowledgements

None.

## Ethical Statements

The study protocol was approved by the Joint Chinese University of Hong Kong–New Territories East Cluster Clinical Research Ethics Committee (CREC 2021.475).

## Conflicts of Interest

Raymond S. Y. Tang is a consultant for Olympus Corporation and Boston Scientific. Anthony Y. B. Teoh is a consultant for Boston Scientific, Cook Medical, Taewoong, Microtech, MI Tech, and CMR Medical Corporations. Terry C. F. Yip has served as an advisory committee member, grant recipient, and speaker for Gilead Sciences. The remaining authors declare that they have no financial conflict of interest with regard to the content of this report.

## Author Contributions

All authors have read and approved the final manuscript. T. Y. Chua designed research, conducted research, wrote the paper, and had primary responsibility for final content. A. Y. B. Teoh, S. M. Chan, T. T. Chan, J. Y. W. Lau, and P. W. Y. Chiu conducted research. T. C. F. Yip analyzed data/performed statistical analysis. R. S. Y. Tang designed research, conducted research, analyzed data/performed statistical analysis, wrote the paper, and had primary responsibility for final content.

## Data availability statement

The data that support the findings of this study are available upon request.
